# Tetra­kis(4-benzoyl­pyridine-κ*N*)bis­(iso­thio­cyanato-κ*N*)manganese(II)

**DOI:** 10.1107/S2056989018016432

**Published:** 2018-11-30

**Authors:** Carsten Wellm, Christian Näther

**Affiliations:** a Institut für Anorganische Chemie, Universität Kiel, Max-Eyth. Str. 2, 241128 Kiel

**Keywords:** crystal structure, mangan(II) thio­cyanate, discrete complex, hydrogen bonding, isotypism

## Abstract

The asymmetric unit of the title compound, [Mn(NCS)_2_(C_12_H_9_NO)_4_], consists of one Mn^II^ cation located on a centre of inversion, one thio­cyanate anion and two 4-benzoyl­pyridine co-ligands. The Mn^II^ cation is octa­hedrally coordinated by two terminally N-bonded anionic ligands and four N-bonded 4-benzoyl­pyridine co-ligands within a slightly distorted octa­hedron. Individual complexes are linked by inter­molecular C—H⋯O hydrogen-bonding inter­actions into chains running along the *c-*axis direction. Simultaneous thermogravimetry and differential scanning calorimetry measurements reveal a decomposition in two separate steps, in each of which two co-ligands are removed. The compound obtained after the first step has the composition [Mn(NCS)_2_(C_12_H_9_NO)_2_] and is of unknown structure, before in the second step decomposition into [Mn(NCS)_2_] is observed. Magnetic susceptibility measurements show the Mn^II^ cations to be in the high-spin state, and that weak anti­ferromagnetic inter­actions between the complexes are present.

## Abstract 2

In the crystal structure of the title compound, the Mn^II^ cations are octa­hedrally coordinated by two terminally N-bonded thio­cyanate anions and four 4-benzoyl­pyridine coligands into discrete complexes, which are further linked into chains by inter­molecular C—H⋯O hydrogen bonding.

## Chemical context   

Thio­cyanate anions are versatile ligands that, in combination with neutral organic co-ligands, can form coordination compounds and polymers of different dimensionality. The most common coordination modes include *N*-terminal and *μ*-1,3-bridging (Buckingham, 1994 ▸; Palion-Gazda *et al.*, 2017 ▸; Mautner *et al.*, 2017 ▸). The bridging mode is of special inter­est because magnetic exchange can be mediated by the anionic ligands (Palion-Gazda *et al.*, 2015 ▸; Mekuimemba *et al.*, 2018 ▸; González *et al.*, 2012 ▸; Guillet *et al.*, 2016 ▸). In this context, we have reported the syntheses, structures and magnetic properties of a number of compounds, in which transition metal cations such as Mn^II^, Fe^II^, Co^II^ and Ni^II^ are octa­hedrally coordinated by two neutral N-donor co-ligands and four thio­cyanate anions and are linked into linear or corrugated chains by pairs of anionic ligands (Suckert *et al.*, 2017**a* ▸;* Werner *et al.*, 2015 ▸; Wöhlert *et al.*, 2013 ▸, 2014**a* ▸,b* ▸). In the course of our project, we have also used 4-benzoyl­pyridine as co-ligand, leading to the formation of two isotypic chain compounds with general composition [*M*(NCS)_2_(4-benzoyl­pyridine)_2_] (*M* = Co, Ni). In both compounds, dominating ferromagnetic inter­actions are observed but the Co^II^ compound additionally shows a slow relaxation of the magnetization, indicating single-chain magnetism (Rams *et al.*, 2017 ▸; Jochim *et al.*, 2018 ▸). In contrast to most other compounds, in which all ligands are in the *trans*-position, in the 4-benzoyl­pyridine coordination polymers with Co^II^ and Ni^II^, the central metal cation shows a *cis*–*cis*–*trans* coordination. However, the corresponding Cd compound [Cd(NCS)_2_(4-benzoyl­pyridine)_2_] shows an all-*trans* coordination of the Cd^II^ cation (Neumann *et al.*, 2018*a* ▸).
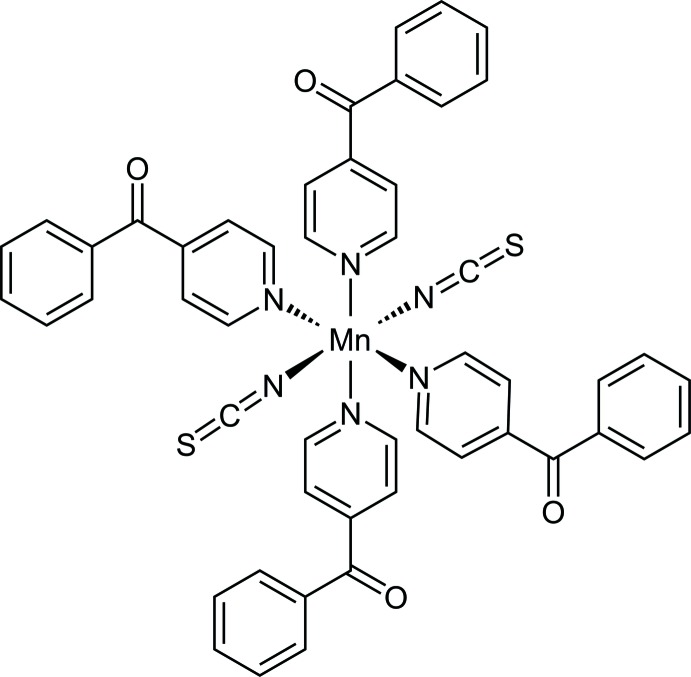


In this context, the question arose about which kind of metal coordination is observed for the corresponding Mn^II^ compound, which is less chalcophilic compared to Co^II^ and Ni^II^. Therefore, [Mn(NCS)_2_] was reacted with 4-benzoyl­pyridine in different ratios and only crystals of a compound with composition [Mn(NCS)_2_(4-benzoyl­pyridine)_4_] were obtained, as determined by single crystal X-ray diffraction. If the experimental X-ray powder pattern is compared with that calculated from single crystal data, it is obvious that a pure crystalline phase has been obtained (see Fig. *S*1 in the supporting information). In the IR spectrum, the asymmetric C≡N-stretching vibration is observed at 2054 cm^−1^, which is in agreement with the presence of terminal N-bonded thio­cyanate anions (Fig. *S*2). Magnetic susceptibility measurements in a field of 1 kOe show paramagnetic behaviour. From the temperature-independent susceptibility curve, it is obvious that dominating anti­ferromagnetic inter­actions are present, which is frequently observed for similar discrete complexes based on [Mn(NCS)_2_]. The susceptibility curve was analysed using the Curie–Weiss law, leading to a magnetic moment of 6.0 µ_B_, which is in good agreement with that expected for an Mn^II^ cation in a high-spin configuration (Fig. *S*3). As previously shown, co-ligand-rich precursor complexes can be transformed into co-ligand-deficient compounds with more condensed thio­cyanate networks by thermal decomposition (Neumann *et al.*, 2018*b* ▸). Therefore, the title compound was investigated by simultaneous thermogravimetry and differential thermoanalysis (TG–DTA). Upon heating, two mass loss steps are observed in the TG curve, accompanied by two endothermic events in the DTA curve (Fig. *S*4). The experimental mass loss in each step of 40.4 and 40.5% is in good agreement with that calculated for the removal of two 4-benzoyl­pyridine ligands in each step. When in a second TG measurement the residue formed after the first mass loss was isolated and investigated by X-ray powder diffraction, it became clear that the powder pattern was not related to those for [Co(NCS)_2_(4-benzoyl­pyridine)_2_] and [Cd(NCS)_2_(4-benz­oyl­pyridine)_2_], indicating that a new crystalline phase had formed (Fig. *S*5). Indexing of the powder pattern failed, and therefore the structure of this compound remains unknown. However, the C≡N stretch observed in the IR spectrum of this residue is found at 2078 cm^−1^, which is close to that in [Cd(NCS)_2_(4-benzoyl­pyridine)_2_] (2088 cm^−1^), indicating the presence of *μ*-1,3-bridging anionic ligands (Fig. *S*6).

## Structural commentary   

**Figure 1:**
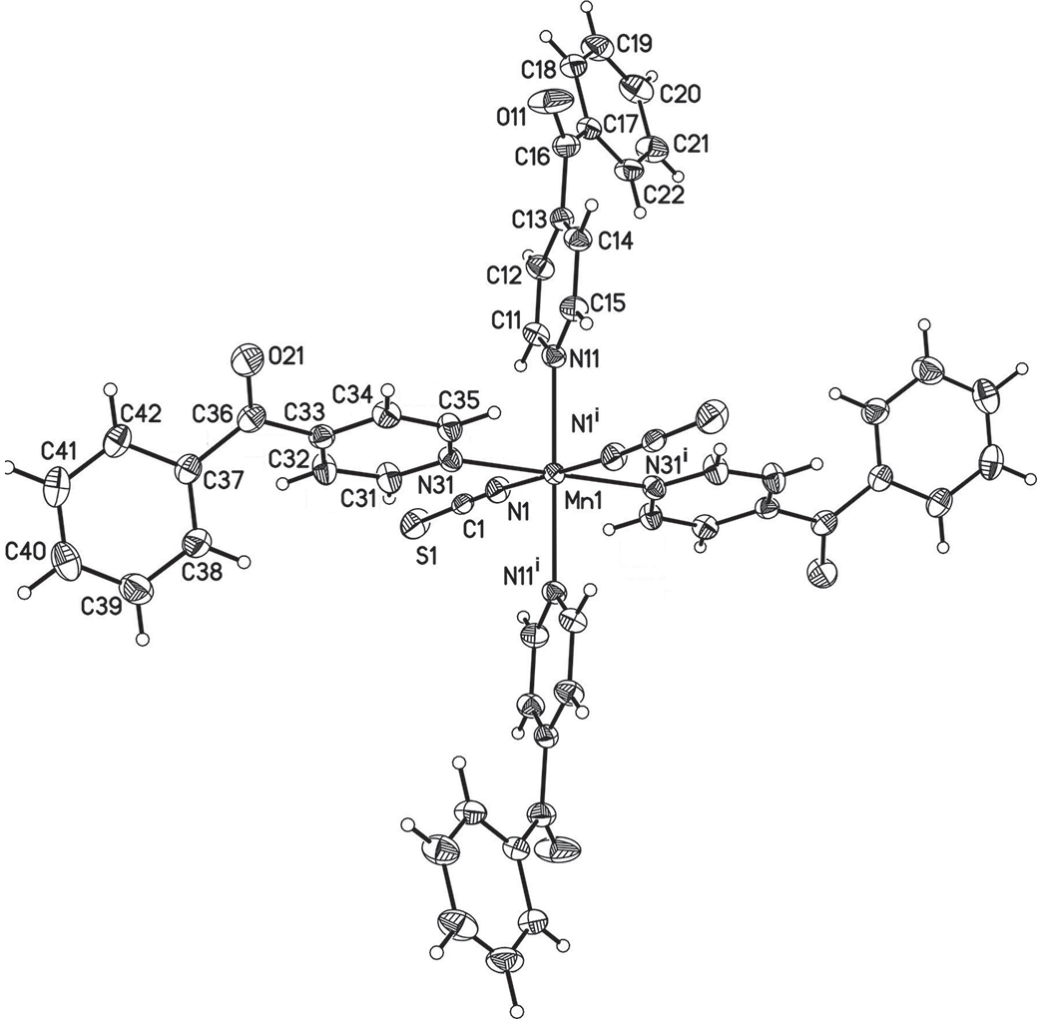
View of a discrete complex with the atom labelling and displacement ellipsoids drawn at the 50% probability level. [Symmetry code: (i) −*x*, −*y* + 1, −*z* + 1.]

In the crystal structure of the title compound, the Mn^II^ cations are located on centers of inversion, whereas the unique thio­cyanate anion and the two crystallographically independent 4-benzoyl­pyridine co-ligands occupy general positions. The Mn^II^ cation is ocahedrally coordinated by two N-bonded terminal thio­cyanate anions and four neutral N-bonded 4-benzoyl­pyridine ligands. The Mn—N bond lengths are considerably shorter for the anionic ligand [2.1658 (15) Å] than those for the neutral co-ligands [2.3200 (14) and 2.3232 (14) Å; Fig. 1 ▸ and Table 1 ▸]. The bond lengths and angles reveal a slight distortion of the MnN_6_ octa­hedron (Table 1 ▸), which is also obvious from the angle variance of 4.8 and the quadratic elongation of 1.022 (Robinson *et al.*, 1971 ▸). Neither the pyridine nor the phenyl rings of the two 4-benzoyl­pyridine ligands are coplanar with the carbonyl planes. In the first ligand, the phenyl plane (C17–C22) is inclined at an angle of 23.08 (11)° to the plane of the carbonyl group (O11,C13,C16,C17) and to the pyridine plane (N11,C11–C15) by 37.33 (10)°. Corresponding values for the second co-ligand are 24.07 (11)° between the carbonyl plane (O21,C33,C36,C37) and the phenyl ring (C37–C42) and 36.58 (10)° for the pyridine ring (N31,C31–C35). There are weak intra­molecular C—H⋯N inter­actions between some of the aromatic hydrogen atoms and the thio­cyanate N atoms, which might contribute to the stabilization of the conformation of the complex (Table 2 ▸).

## Supra­molecular features   

**Figure 2:**
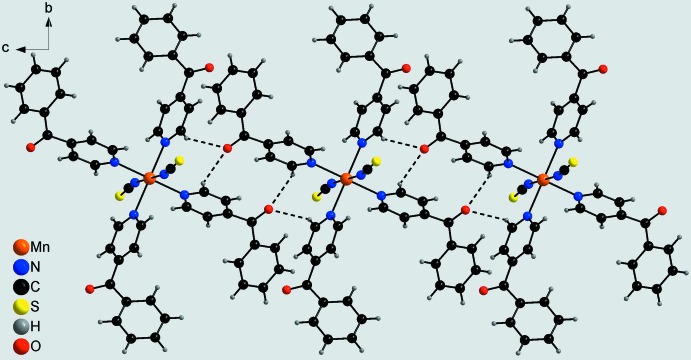
Crystal structure of the title compound showing a chain formed by inter­molecular C—H⋯O hydrogen bonding (dashed lines).

**Figure 3:**
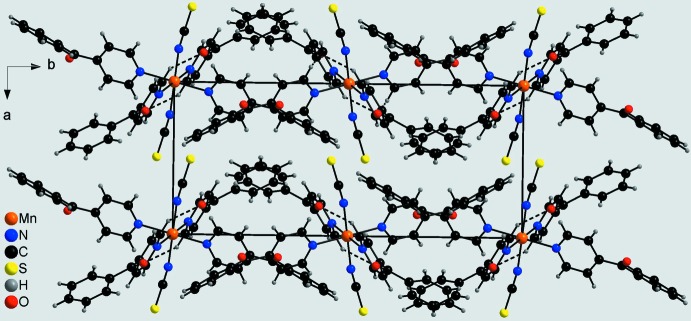
Crystal structure of the title compound in a view along the *c* axis. Inter­molecular C—H⋯O hydrogen bonds are shown as dashed lines.

In the crystal structure of the title compound, discrete complexes are linked by inter­molecular C—H⋯O hydrogen-bonding inter­actions between the carbonyl O atom and the two hydrogen atoms H15 and H35. Each complex forms four such hydrogen bonds to neighbouring complexes, leading to the formation of chains that elongate in the direction of the *c* axis (Figs. 2 ▸ and 3 ▸, Table 2 ▸). Between the chains no distinct inter­molecular inter­actions apart from van der Waals inter­actions are observed (Fig. 3 ▸).

## Database survey   

In the Cambridge Structure Database (Version 5.39, last update Aug 2018; Groom *et al.*, 2016 ▸), there are ten structures of coordination compounds reported that are comprised of 4-benzoyl­pyridine ligands, thio­cyanate anions and different transition metal cations. Firstly, there are two complexes in which the cations are coordinated each by two terminal N-bonded thio­cyanate anions and two 4-benzoyl­pyridine ligands to form a square-planar complex with Cu^II^ (Bai *et al.*, 2011 ▸) and a tetra­hedral complex with Zn^II^ (Neumann *et al.*, 2018*a* ▸). There are also two complexes with coordinating solvate ligands, in which the Co^II^ cation is octa­hedrally coordinated by two terminal N-bonded thio­cyanate anions, two 4-benzoyl­pyridine ligands and either two methanol (Suckert *et al.*, 2017*a* ▸), or two aceto­nitrile mol­ecules (Suckert *et al.*, 2017*b* ▸). As mentioned above, there is also a chain compound with composition [Co(NCS)_2_(4-benzoyl­pyridine)_2_] in which the Co^II^ cations are linked by pairs of *μ*-1,3-coordinating thio­cyanate anions (Rams *et al.*, 2017 ▸). It is also noted that two additional chain compounds with Cd^II^ and Ni^II^ are reported in literature (Jochim *et al.*, 2018 ▸; Neumann *et al.*, 2018*a* ▸). Finally, there are one Ni^II^ (Soliman *et al.*, 2014 ▸), one Co^II^ (Drew *et al.*,1985 ▸), one Zn and one Cd compound (Neumann *et al.*, 2018*a* ▸) that are isotypic with the title complex.

## Synthesis and crystallization   

Ba(SCN)_2_·3H_2_O and 4-benzoyl­pyridine were purchased from Alfa Aesar. Mn(SO_4_)·4H_2_O was purchased from Merck. All solvents and reactants were used without further purification. Mn(NCS)_2_ was prepared by the reaction of equimolar amounts of MnSO_4_·4H_2_O and Ba(NCS)_2_·3H_2_O in water. The resulting white precipitate of BaSO_4_ was filtered off, and the solvent was evaporated from the filtrate. The product was finally dried at room-temperature.

Crystals of the title compound suitable for single crystal X-ray diffraction were obtained by the reaction of 51.3 mg Mn(NCS)_2_ (0.30 mmol) with 27.5 mg 4-benzoyl­pyridine (0.15 mmol) in methanol (1.5 mL) within three days.

## Refinement   

Crystal data, data collection and structure refinement details are summarized in Table 3 ▸. Hydrogen atoms were positioned with idealized geometry (C—H = 0.95 Å) and were refined with *U*_iso_(H) = 1.2 *U*_eq_(C) using a riding model.

## Supplementary Material

Crystal structure: contains datablock(s) I. DOI: 10.1107/S2056989018016432/wm5475sup1.cif

Structure factors: contains datablock(s) I. DOI: 10.1107/S2056989018016432/wm5475Isup2.hkl

Figure S1. Experimental (top) and calculated X-ray powder pattern (bottom) of the title compound.. DOI: 10.1107/S2056989018016432/wm5475sup3.tif

Figure S2. IR spectrum of the title compound. Given is the value of the CN-stretching vibration.. DOI: 10.1107/S2056989018016432/wm5475sup4.tif

Figure S3. Magnetic susceptibility and inverse susceptibility (inset) as function of temperature for the title compound.. DOI: 10.1107/S2056989018016432/wm5475sup5.tif

Figure S4. TG-DTA curve of the title compound measured with 1%C/min in an nitrogen atmosphere.. DOI: 10.1107/S2056989018016432/wm5475sup6.tif

Figure S5. Experimental X-ray powder pattern of the residues obtained after the first mass loss at 1°C/min, 4°C/min, 8°C/min in the TG-DTA measurement of the title compound together with the patterns calculated for [Cd(SCN)2(4-benzoylpyridine)2] (A) and [Cd(SCN)2(4-Benzoylpyridine)2 (B) retrieved from literature.. DOI: 10.1107/S2056989018016432/wm5475sup7.tif

Figure S6. IR spectrum of the residue obtained after the first mass loss at 1°C. Given is the value of the CN-stretching vibration.. DOI: 10.1107/S2056989018016432/wm5475sup8.tif

CCDC reference: 1879856

Additional supporting information:  crystallographic information; 3D view; checkCIF report

## Figures and Tables

**Table 1:** Selected geometric parameters (Å, °)

Mn1—N1	2.1658 (15)	Mn1—N11	2.3232 (14)
Mn1—N31	2.3200 (14)		
			
N1—Mn1—N31^i^	90.09 (5)	N1^i^—Mn1—N11	88.65 (5)
N1—Mn1—N31	89.91 (5)	N31^i^—Mn1—N11	92.63 (5)
N1—Mn1—N11	91.35 (5)	N31—Mn1—N11	87.37 (5)

Symmetry code: (i) 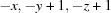.

**Table 2:** Hydrogen-bond geometry (Å, °)

*D*—H⋯*A*	*D*—H	H⋯*A*	*D*⋯*A*	*D*—H⋯*A*
C11—H11⋯N1	0.95	2.57	3.215 (2)	126
C15—H15⋯N1^i^	0.95	2.61	3.195 (2)	120
C15—H15⋯O21^ii^	0.95	2.54	3.263 (2)	133
C31—H31⋯N1	0.95	2.66	3.251 (2)	120
C35—H35⋯N1^i^	0.95	2.56	3.181 (2)	124
C35—H35⋯O21^ii^	0.95	2.63	3.350 (2)	133

Symmetry codes: (i) 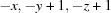; (ii) 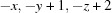.

**Table 3:** Experimental details

Crystal data	
Chemical formula	[Mn(NCS)_2_(C_12_H_9_NO)_4_]
*M*_r_	903.91
Crystal system, space group	Monoclinic, *P*2_1_/*c*
Temperature (K)	200
*a*, *b*, *c* (Å)	9.1463 (6), 20.9990 (11), 11.2177 (7)
β (°)	90.493 (7)
*V* (Å^3^)	2154.4 (2)
*Z*	2
Radiation type	Mo *K*α
μ (mm^−1^)	0.46
Crystal size (mm)	0.12 × 0.03 × 0.03

Data collection	
Diffractometer	Stoe IPDS1
Absorption correction	Numerical (*X-SHAPE* and *X-RED32*; Stoe, 2008 ▸)
*T*_min_, *T*_max_	0.836, 0.989
No. of measured, independent and observed [*I* > 2σ(*I*)] reflections	22833, 4717, 3912
*R*_int_	0.037
(sin θ/λ)_max_ (Å^−1^)	0.639

Refinement	
*R*[*F* ^2^ > 2σ(*F* ^2^)], *wR*(*F* ^2^), *S*	0.040, 0.109, 1.04
No. of reflections	4717
No. of parameters	287
H-atom treatment	H-atom parameters constrained
Δρ_max_, Δρ_min_ (e Å^−3^)	0.48, −0.42

Computer programs: *X-AREA* (Stoe, 2008 ▸), *SHELXS97* (Sheldrick, 2008 ▸), *SHELXL2014/7* (Sheldrick, 2015 ▸), *XP* in *SHELXTL* (Sheldrick, 2008 ▸), *DIAMOND* (Brandenburg, 1999 ▸) and *publCIF* (Westrip, 2010 ▸).

## supplementary crystallographic information

### Crystal data

**Table:**

[Mn(NCS)_2_(C_12_H_9_NO)_4_]	*F*(000) = 934
*M**_r_* = 903.91	*D*_x_ = 1.393 Mg m^−^^3^
Monoclinic, *P*2_1_/*c*	Mo *K*α radiation, λ = 0.71073 Å
*a* = 9.1463 (6) Å	Cell parameters from 22833 reflections
*b* = 20.9990 (11) Å	θ = 2.4–27.0°
*c* = 11.2177 (7) Å	µ = 0.46 mm^−^^1^
β = 90.493 (7)°	*T* = 200 K
*V* = 2154.4 (2) Å^3^	Needle, colorless
*Z* = 2	0.12 × 0.03 × 0.03 mm

### Data collection

**Table:**

Stoe IPDS-1 diffractometer	3912 reflections with *I* > 2σ(*I*)
Phi scans	*R*_int_ = 0.037
Absorption correction: numerical (*X-Shape* and *X-RED32*; Stoe, 2008)	θ_max_ = 27.0°, θ_min_ = 2.4°
*T*_min_ = 0.836, *T*_max_ = 0.989	*h* = −11→11
22833 measured reflections	*k* = −26→26
4717 independent reflections	*l* = −14→14

### Refinement

**Table:**

Refinement on *F*^2^	Hydrogen site location: inferred from neighbouring sites
Least-squares matrix: full	H-atom parameters constrained
*R*[*F*^2^ > 2σ(*F*^2^)] = 0.040	*w* = 1/[σ^2^(*F*_o_^2^) + (0.0684*P*)^2^ + 0.645*P*] where *P* = (*F*_o_^2^ + 2*F*_c_^2^)/3
*wR*(*F*^2^) = 0.109	(Δ/σ)_max_ < 0.001
*S* = 1.03	Δρ_max_ = 0.48 e Å^−^^3^
4717 reflections	Δρ_min_ = −0.42 e Å^−^^3^
287 parameters	Extinction correction: SHELXL, Fc^*^=kFc[1+0.001xFc^2^λ^3^/sin(2θ)]^-1/4^
0 restraints	Extinction coefficient: 0.0166 (16)

### Special details

**Table:**

Geometry. All esds (except the esd in the dihedral angle between two l.s. planes) are estimated using the full covariance matrix. The cell esds are taken into account individually in the estimation of esds in distances, angles and torsion angles; correlations between esds in cell parameters are only used when they are defined by crystal symmetry. An approximate (isotropic) treatment of cell esds is used for estimating esds involving l.s. planes.

### Fractional atomic coordinates and isotropic or equivalent isotropic displacement parameters (Å^2^)

**Table:**

	*x*	*y*	*z*	*U*_iso_*/*U*_eq_	
Mn1	0.0000	0.5000	0.5000	0.01849 (12)	
N1	0.21622 (16)	0.51148 (7)	0.42577 (14)	0.0257 (3)	
C1	0.33230 (18)	0.52654 (8)	0.39340 (14)	0.0212 (3)	
S1	0.49322 (5)	0.54834 (3)	0.34865 (5)	0.03905 (16)	
N11	0.06243 (16)	0.40128 (7)	0.57884 (13)	0.0222 (3)	
C11	0.18424 (19)	0.37239 (8)	0.54236 (16)	0.0245 (4)	
H11	0.2472	0.3948	0.4901	0.029*	
C12	0.2235 (2)	0.31115 (9)	0.57705 (17)	0.0276 (4)	
H12	0.3101	0.2920	0.5475	0.033*	
C13	0.1348 (2)	0.27844 (8)	0.65524 (15)	0.0240 (4)	
C14	0.0115 (2)	0.30940 (9)	0.69848 (16)	0.0267 (4)	
H14	−0.0492	0.2892	0.7554	0.032*	
C15	−0.0217 (2)	0.36995 (9)	0.65760 (16)	0.0262 (4)	
H15	−0.1073	0.3903	0.6862	0.031*	
C16	0.1712 (2)	0.21249 (9)	0.69836 (17)	0.0301 (4)	
C17	0.2360 (2)	0.16528 (8)	0.61533 (17)	0.0262 (4)	
C18	0.3095 (2)	0.11280 (9)	0.66421 (19)	0.0323 (4)	
H18	0.3239	0.1100	0.7480	0.039*	
C19	0.3611 (2)	0.06508 (10)	0.5909 (2)	0.0396 (5)	
H19	0.4118	0.0298	0.6243	0.047*	
C20	0.3392 (2)	0.06865 (10)	0.4687 (2)	0.0397 (5)	
H20	0.3742	0.0356	0.4186	0.048*	
C21	0.2664 (2)	0.12032 (10)	0.41936 (19)	0.0368 (5)	
H21	0.2517	0.1226	0.3355	0.044*	
C22	0.2148 (2)	0.16883 (9)	0.49211 (17)	0.0298 (4)	
H22	0.1652	0.2043	0.4581	0.036*	
O11	0.1455 (2)	0.19888 (8)	0.80158 (13)	0.0540 (5)	
N31	0.08153 (16)	0.54430 (7)	0.67799 (12)	0.0225 (3)	
C31	0.2075 (2)	0.57669 (9)	0.68841 (16)	0.0291 (4)	
H31	0.2660	0.5819	0.6195	0.035*	
C32	0.2566 (2)	0.60297 (10)	0.79556 (16)	0.0292 (4)	
H32	0.3455	0.6264	0.7989	0.035*	
C33	0.17349 (19)	0.59438 (8)	0.89755 (15)	0.0230 (3)	
C34	0.0450 (2)	0.55967 (9)	0.88747 (15)	0.0254 (4)	
H34	−0.0136	0.5522	0.9556	0.030*	
C35	0.00293 (19)	0.53596 (9)	0.77729 (16)	0.0256 (4)	
H35	−0.0859	0.5127	0.7717	0.031*	
C36	0.2209 (2)	0.61668 (9)	1.01957 (15)	0.0266 (4)	
C37	0.2974 (2)	0.67860 (9)	1.03561 (15)	0.0258 (4)	
C38	0.2813 (2)	0.72874 (10)	0.95543 (18)	0.0349 (4)	
H38	0.2243	0.7231	0.8850	0.042*	
C39	0.3481 (3)	0.78705 (10)	0.9779 (2)	0.0403 (5)	
H39	0.3353	0.8214	0.9236	0.048*	
C40	0.4330 (2)	0.79497 (11)	1.0789 (2)	0.0376 (5)	
H40	0.4799	0.8346	1.0937	0.045*	
C41	0.4501 (2)	0.74516 (11)	1.15904 (19)	0.0387 (5)	
H41	0.5086	0.7508	1.2286	0.046*	
C42	0.3826 (2)	0.68749 (10)	1.13822 (17)	0.0325 (4)	
H42	0.3941	0.6537	1.1938	0.039*	
O21	0.19390 (18)	0.58258 (8)	1.10490 (12)	0.0414 (4)	

### Atomic displacement parameters (Å^2^)

**Table:**

	*U*^11^	*U*^22^	*U*^33^	*U*^12^	*U*^13^	*U*^23^
Mn1	0.01830 (19)	0.01812 (19)	0.01906 (19)	−0.00066 (12)	0.00066 (13)	−0.00002 (13)
N1	0.0184 (7)	0.0311 (8)	0.0276 (8)	−0.0008 (6)	0.0024 (6)	0.0024 (6)
C1	0.0247 (8)	0.0201 (8)	0.0188 (7)	0.0036 (6)	−0.0038 (6)	0.0001 (6)
S1	0.0221 (2)	0.0553 (3)	0.0398 (3)	−0.0059 (2)	0.00310 (19)	0.0146 (2)
N11	0.0260 (7)	0.0180 (7)	0.0226 (7)	0.0007 (5)	0.0002 (5)	−0.0015 (5)
C11	0.0269 (9)	0.0198 (8)	0.0269 (8)	−0.0004 (6)	0.0044 (7)	0.0003 (7)
C12	0.0277 (9)	0.0218 (9)	0.0333 (9)	0.0029 (7)	0.0045 (7)	−0.0003 (7)
C13	0.0308 (9)	0.0197 (8)	0.0214 (8)	−0.0013 (6)	−0.0036 (7)	−0.0007 (6)
C14	0.0318 (9)	0.0251 (9)	0.0231 (8)	−0.0024 (7)	0.0032 (7)	0.0030 (7)
C15	0.0277 (9)	0.0246 (9)	0.0263 (8)	0.0022 (7)	0.0033 (7)	0.0013 (7)
C16	0.0411 (11)	0.0234 (9)	0.0259 (9)	0.0002 (7)	−0.0043 (8)	0.0024 (7)
C17	0.0284 (9)	0.0191 (8)	0.0312 (9)	−0.0009 (6)	−0.0020 (7)	0.0025 (7)
C18	0.0320 (10)	0.0262 (9)	0.0385 (10)	0.0010 (7)	−0.0067 (8)	0.0070 (8)
C19	0.0320 (10)	0.0263 (10)	0.0604 (14)	0.0095 (8)	0.0012 (9)	0.0071 (9)
C20	0.0407 (11)	0.0270 (10)	0.0516 (13)	0.0072 (8)	0.0128 (10)	−0.0026 (9)
C21	0.0480 (12)	0.0283 (10)	0.0343 (10)	0.0037 (8)	0.0079 (9)	−0.0010 (8)
C22	0.0377 (10)	0.0216 (9)	0.0302 (9)	0.0044 (7)	0.0008 (8)	0.0032 (7)
O11	0.1033 (15)	0.0334 (8)	0.0253 (7)	0.0149 (9)	0.0048 (8)	0.0063 (6)
N31	0.0257 (7)	0.0212 (7)	0.0206 (7)	−0.0026 (5)	0.0019 (5)	−0.0021 (5)
C31	0.0318 (9)	0.0354 (10)	0.0201 (8)	−0.0108 (8)	0.0050 (7)	−0.0030 (7)
C32	0.0293 (9)	0.0354 (10)	0.0231 (8)	−0.0102 (7)	0.0025 (7)	−0.0048 (7)
C33	0.0276 (8)	0.0209 (8)	0.0205 (8)	0.0010 (6)	−0.0002 (6)	0.0002 (6)
C34	0.0284 (9)	0.0270 (9)	0.0209 (8)	−0.0007 (7)	0.0051 (7)	0.0004 (7)
C35	0.0245 (8)	0.0278 (9)	0.0244 (8)	−0.0047 (7)	0.0017 (7)	−0.0020 (7)
C36	0.0285 (9)	0.0315 (9)	0.0197 (8)	0.0028 (7)	0.0012 (7)	0.0003 (7)
C37	0.0261 (9)	0.0310 (9)	0.0204 (8)	0.0022 (7)	0.0009 (7)	−0.0035 (7)
C38	0.0445 (12)	0.0312 (10)	0.0287 (10)	0.0012 (8)	−0.0082 (8)	−0.0006 (8)
C39	0.0524 (13)	0.0292 (10)	0.0393 (11)	−0.0004 (9)	0.0001 (10)	−0.0017 (9)
C40	0.0341 (10)	0.0375 (11)	0.0415 (11)	−0.0050 (8)	0.0075 (9)	−0.0157 (9)
C41	0.0327 (10)	0.0515 (13)	0.0317 (10)	−0.0031 (9)	−0.0046 (8)	−0.0113 (9)
C42	0.0340 (10)	0.0410 (11)	0.0224 (9)	0.0018 (8)	−0.0041 (7)	−0.0028 (8)
O21	0.0565 (10)	0.0459 (9)	0.0218 (7)	−0.0123 (7)	−0.0017 (6)	0.0055 (6)

### Geometric parameters (Å, º)

**Table:**

Mn1—N1	2.1658 (15)	C20—H20	0.9500
Mn1—N1^i^	2.1658 (15)	C21—C22	1.390 (3)
Mn1—N31^i^	2.3200 (14)	C21—H21	0.9500
Mn1—N31	2.3200 (14)	C22—H22	0.9500
Mn1—N11	2.3232 (14)	N31—C35	1.342 (2)
Mn1—N11^i^	2.3232 (14)	N31—C31	1.343 (2)
N1—C1	1.169 (2)	C31—C32	1.393 (2)
C1—S1	1.6249 (18)	C31—H31	0.9500
N11—C11	1.336 (2)	C32—C33	1.391 (3)
N11—C15	1.348 (2)	C32—H32	0.9500
C11—C12	1.390 (3)	C33—C34	1.386 (3)
C11—H11	0.9500	C33—C36	1.507 (2)
C12—C13	1.383 (3)	C34—C35	1.384 (2)
C12—H12	0.9500	C34—H34	0.9500
C13—C14	1.392 (3)	C35—H35	0.9500
C13—C16	1.503 (2)	C36—O21	1.222 (2)
C14—C15	1.385 (3)	C36—C37	1.487 (3)
C14—H14	0.9500	C37—C38	1.392 (3)
C15—H15	0.9500	C37—C42	1.397 (2)
C16—O11	1.218 (2)	C38—C39	1.391 (3)
C16—C17	1.487 (3)	C38—H38	0.9500
C17—C22	1.396 (3)	C39—C40	1.379 (3)
C17—C18	1.400 (3)	C39—H39	0.9500
C18—C19	1.382 (3)	C40—C41	1.387 (3)
C18—H18	0.9500	C40—H40	0.9500
C19—C20	1.386 (3)	C41—C42	1.378 (3)
C19—H19	0.9500	C41—H41	0.9500
C20—C21	1.386 (3)	C42—H42	0.9500
			
N1—Mn1—N1^i^	180.0	C19—C20—H20	119.9
N1—Mn1—N31^i^	90.09 (5)	C21—C20—H20	119.9
N1^i^—Mn1—N31^i^	89.91 (5)	C20—C21—C22	120.3 (2)
N1—Mn1—N31	89.91 (5)	C20—C21—H21	119.9
N1^i^—Mn1—N31	90.09 (5)	C22—C21—H21	119.9
N31^i^—Mn1—N31	180.0	C21—C22—C17	119.80 (18)
N1—Mn1—N11	91.35 (5)	C21—C22—H22	120.1
N1^i^—Mn1—N11	88.65 (5)	C17—C22—H22	120.1
N31^i^—Mn1—N11	92.63 (5)	C35—N31—C31	117.34 (15)
N31—Mn1—N11	87.37 (5)	C35—N31—Mn1	119.43 (11)
N1—Mn1—N11^i^	88.65 (5)	C31—N31—Mn1	123.19 (12)
N1^i^—Mn1—N11^i^	91.35 (5)	N31—C31—C32	123.08 (17)
N31^i^—Mn1—N11^i^	87.37 (5)	N31—C31—H31	118.5
N31—Mn1—N11^i^	92.63 (5)	C32—C31—H31	118.5
N11—Mn1—N11^i^	180.0	C33—C32—C31	118.93 (17)
C1—N1—Mn1	169.93 (14)	C33—C32—H32	120.5
N1—C1—S1	179.33 (17)	C31—C32—H32	120.5
C11—N11—C15	117.48 (15)	C34—C33—C32	118.03 (16)
C11—N11—Mn1	119.44 (11)	C34—C33—C36	118.34 (16)
C15—N11—Mn1	123.04 (12)	C32—C33—C36	123.48 (16)
N11—C11—C12	123.26 (17)	C35—C34—C33	119.41 (16)
N11—C11—H11	118.4	C35—C34—H34	120.3
C12—C11—H11	118.4	C33—C34—H34	120.3
C13—C12—C11	119.06 (17)	N31—C35—C34	123.17 (16)
C13—C12—H12	120.5	N31—C35—H35	118.4
C11—C12—H12	120.5	C34—C35—H35	118.4
C12—C13—C14	118.06 (16)	O21—C36—C37	121.05 (17)
C12—C13—C16	122.15 (17)	O21—C36—C33	118.07 (17)
C14—C13—C16	119.73 (17)	C37—C36—C33	120.88 (15)
C15—C14—C13	119.30 (17)	C38—C37—C42	119.12 (18)
C15—C14—H14	120.4	C38—C37—C36	122.43 (16)
C13—C14—H14	120.4	C42—C37—C36	118.37 (17)
N11—C15—C14	122.72 (17)	C39—C38—C37	120.35 (18)
N11—C15—H15	118.6	C39—C38—H38	119.8
C14—C15—H15	118.6	C37—C38—H38	119.8
O11—C16—C17	121.37 (17)	C40—C39—C38	119.9 (2)
O11—C16—C13	118.55 (18)	C40—C39—H39	120.0
C17—C16—C13	120.07 (16)	C38—C39—H39	120.0
C22—C17—C18	119.47 (18)	C39—C40—C41	120.1 (2)
C22—C17—C16	122.12 (16)	C39—C40—H40	119.9
C18—C17—C16	118.16 (17)	C41—C40—H40	119.9
C19—C18—C17	120.18 (19)	C42—C41—C40	120.34 (19)
C19—C18—H18	119.9	C42—C41—H41	119.8
C17—C18—H18	119.9	C40—C41—H41	119.8
C18—C19—C20	120.15 (19)	C41—C42—C37	120.18 (19)
C18—C19—H19	119.9	C41—C42—H42	119.9
C20—C19—H19	119.9	C37—C42—H42	119.9
C19—C20—C21	120.1 (2)		

Symmetry code: (i) −*x*, −*y*+1, −*z*+1.

### Hydrogen-bond geometry (Å, º)

**Table:**

*D*—H···*A*	*D*—H	H···*A*	*D*···*A*	*D*—H···*A*
C11—H11···N1	0.95	2.57	3.215 (2)	126
C15—H15···N1^i^	0.95	2.61	3.195 (2)	120
C15—H15···O21^ii^	0.95	2.54	3.263 (2)	133
C31—H31···N1	0.95	2.66	3.251 (2)	120
C35—H35···N1^i^	0.95	2.56	3.181 (2)	124
C35—H35···O21^ii^	0.95	2.63	3.350 (2)	133

Symmetry codes: (i) −*x*, −*y*+1, −*z*+1; (ii) −*x*, −*y*+1, −*z*+2.
